# Erratum for Versluis et al., “A Multiscale Spatiotemporal Model Including a Switch from Aerobic To Anaerobic Metabolism Reproduces Succession in the Early Infant Gut Microbiota”

**DOI:** 10.1128/msystems.01150-22

**Published:** 2022-12-12

**Authors:** David M. Versluis, Ruud Schoemaker, Ellen Looijesteijn, Daniël Muysken, Prescilla V. Jeurink, Marcel Paques, Jan M. W. Geurts, Roeland M. H. Merks

## ERRATUM

Volume 7, no. 5, e00446-22, 2022 , https://doi.org/10.1128/mSystems.00446-22. Page 19: Equation 3 should read as follows:

$${\vec{F}}_{\text{ub}}\left(\vec{x},t\right)=\frac{\vec{c}\left(\vec{x},t\right)}{B\left(\vec{x},t\right)}$$

where $\vec{c}(\vec{x},t)$ is the vector of metabolite concentrations at point $\vec{x}$, $\vec{x}$ indicates the location, and $B(\vec{x},t)$ is the size of the local bacterial population in population units.

Page 20: Equation 5 should read as follows:

$$\frac{d\vec{c}\left(\vec{x},t\right)}{\mathrm{dt}}={\vec{F}}_{\text{out}}\left(\vec{x},t\right)B\left(\vec{x},t\right)-{\vec{F}}_{\text{in}}\left(\vec{x},t\right)B\left(\vec{x},t\right)+\;\frac{D}{L^{2}}\underset{\vec{i}\in \mathrm{NB}\left(\vec{x}\right)}{\sum }\left(\vec{c}\left[\vec{i},t\right]-\vec{c}\left[\vec{x},t\right]\right)$$

Page 20: Equation 6 should read as follows:

$$\frac{\mathrm{dB}\left(\vec{x},t\right)}{\mathrm{dt}}=B\left(\vec{x},t\right)g\left(\vec{x},t\right)$$

