# Supplementary figures and images for: A Multiscale Spatiotemporal Model Including a Switch from Aerobic to Anaerobic Metabolism Reproduces Succession in the Early Infant Gut Microbiota
Source: mSystems. 2022 Sep 1;7(5):e00446-22. doi: 10.1128/msystems.00446-22 (PMC9600552; doi:10.1128/msystems.00446-22)

A

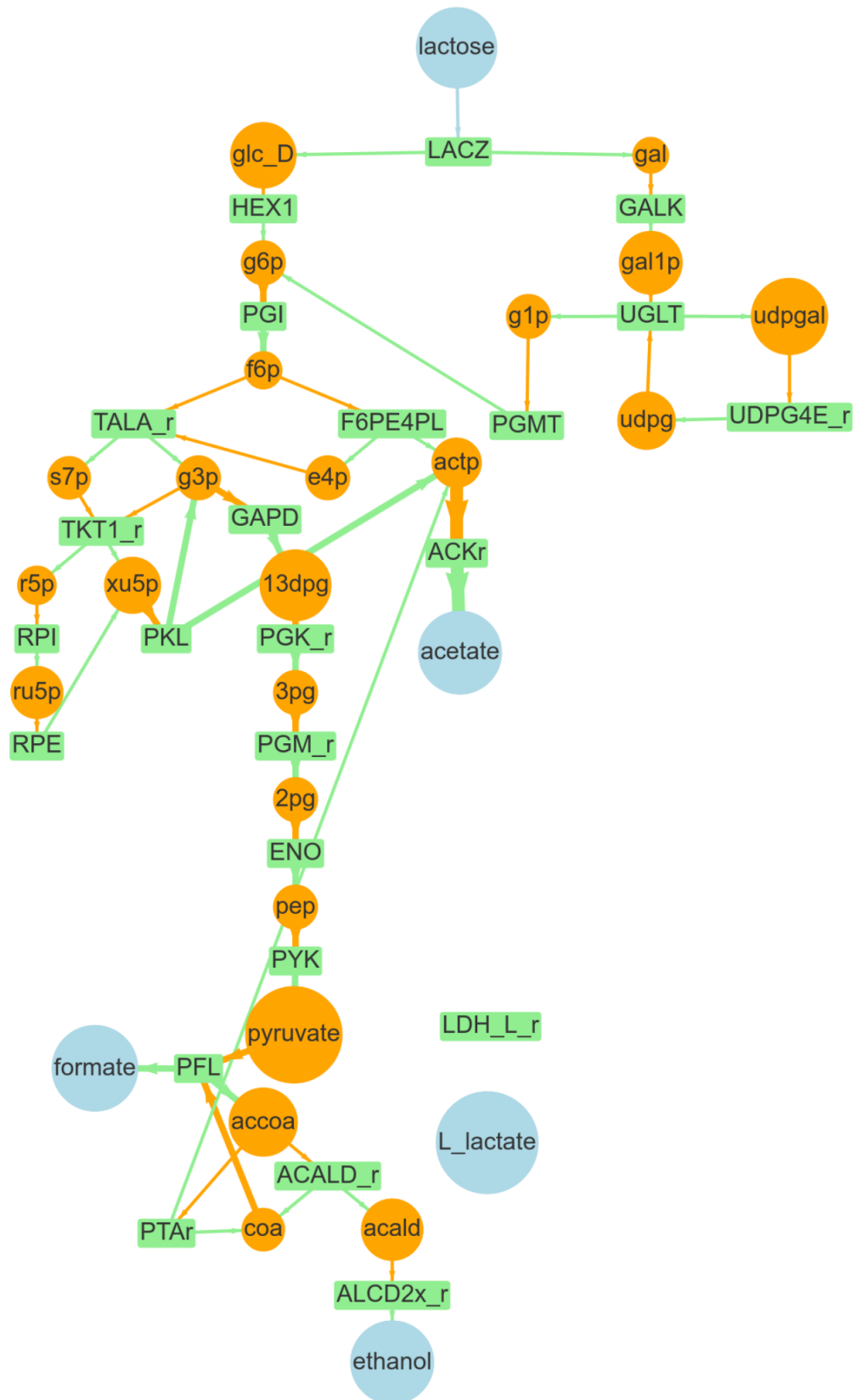

B

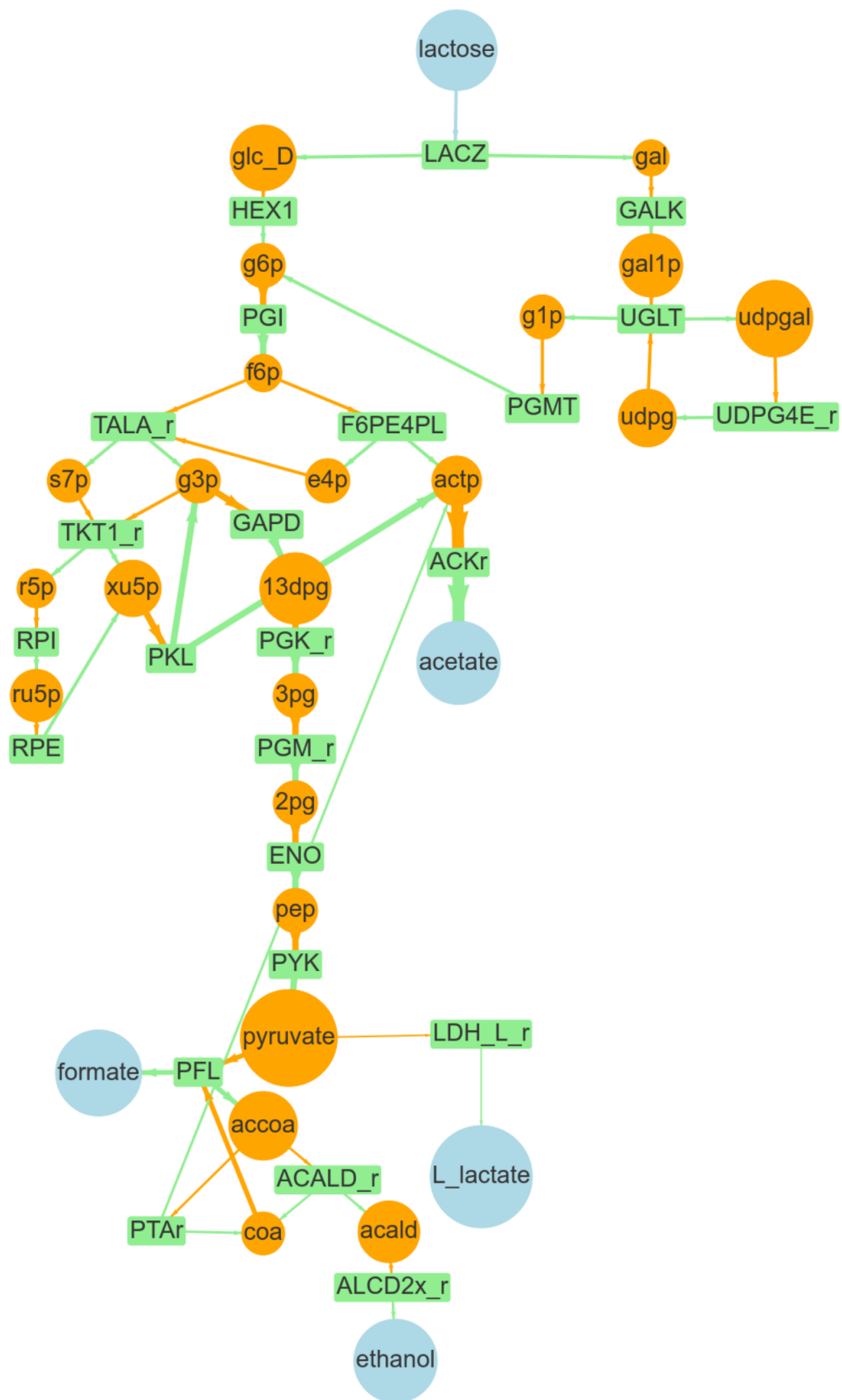

C

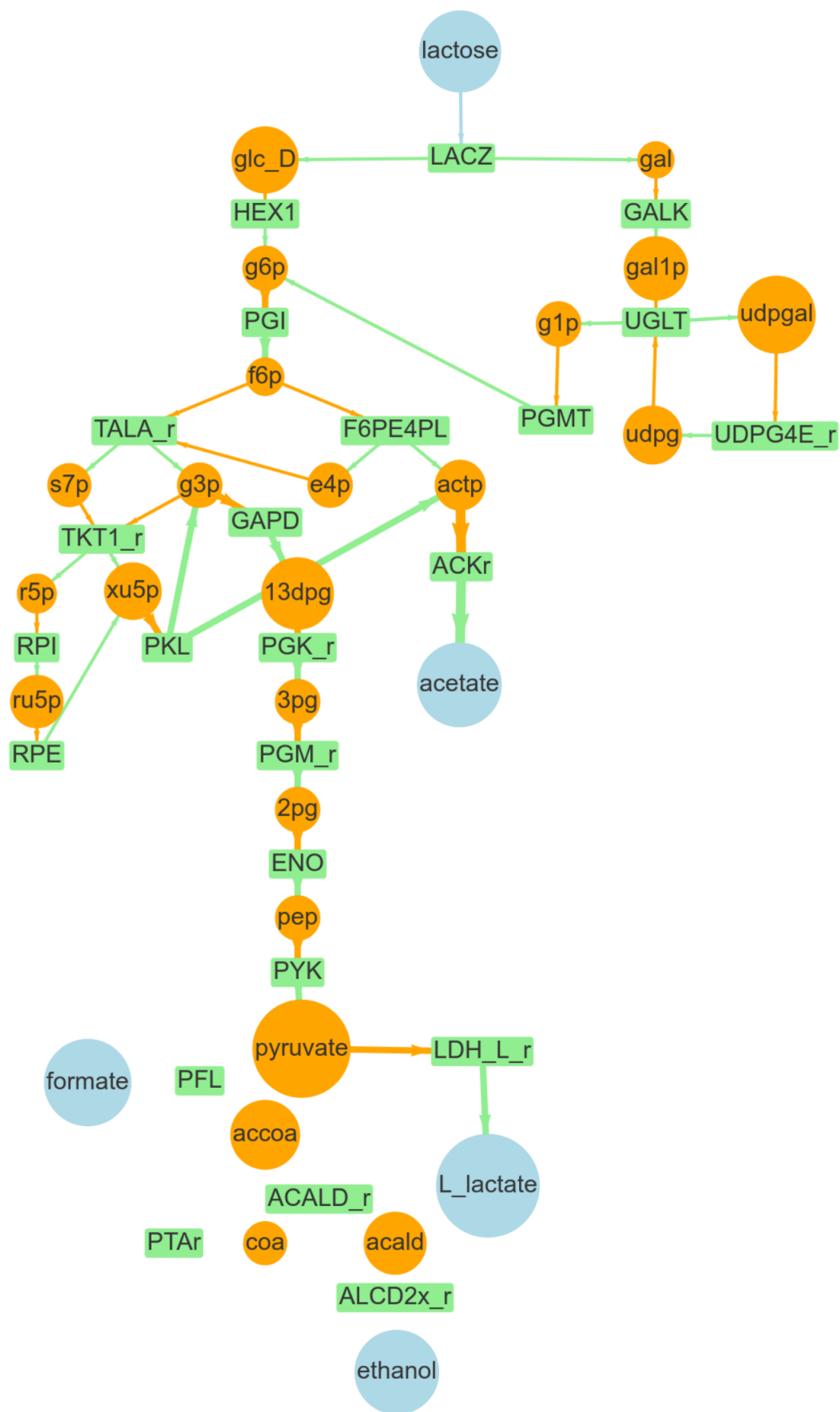

Supplement: FIG S2 [file msystems.00446-22-s0006.pdf]

A

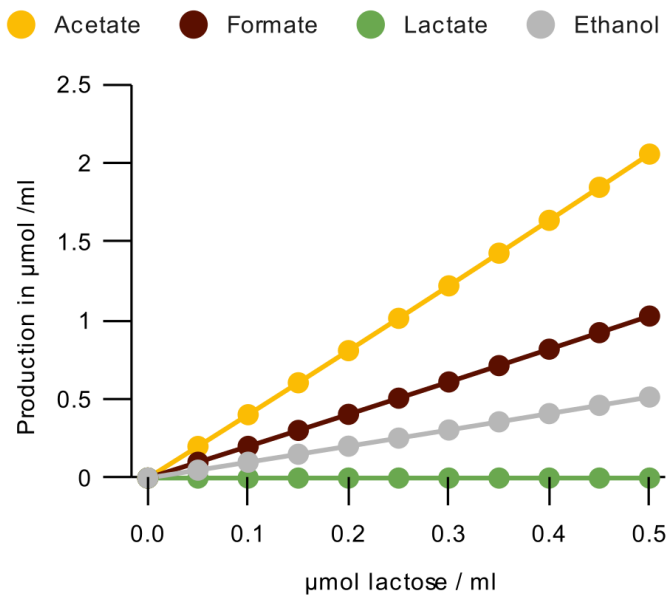

B

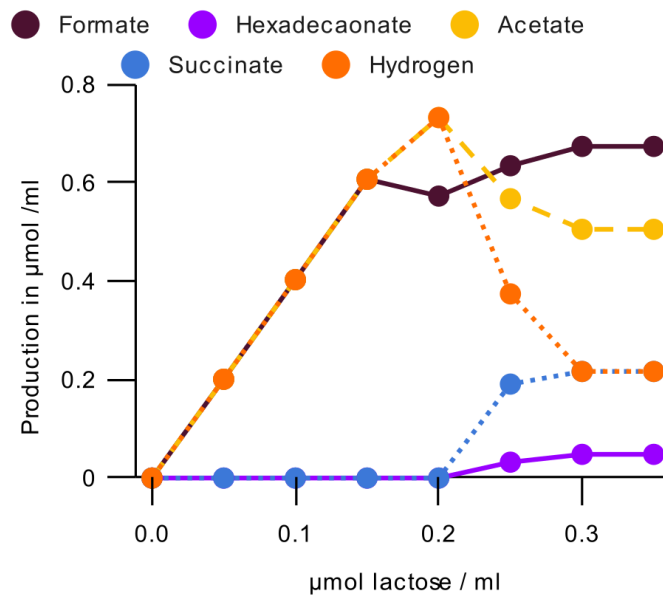

C

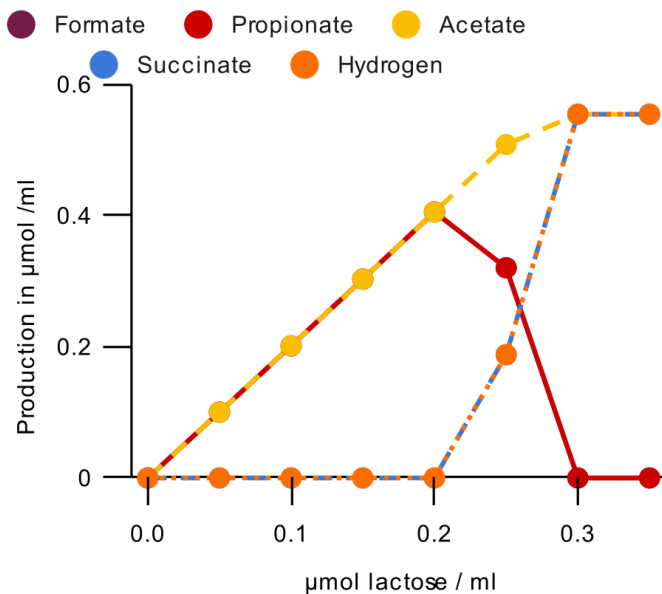

D

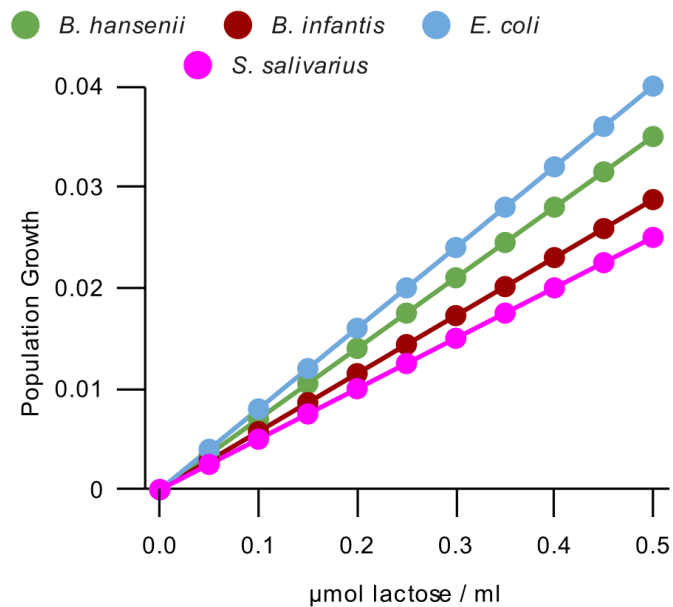

Supplement: FIG S1 [file msystems.00446-22-s0005.pdf]

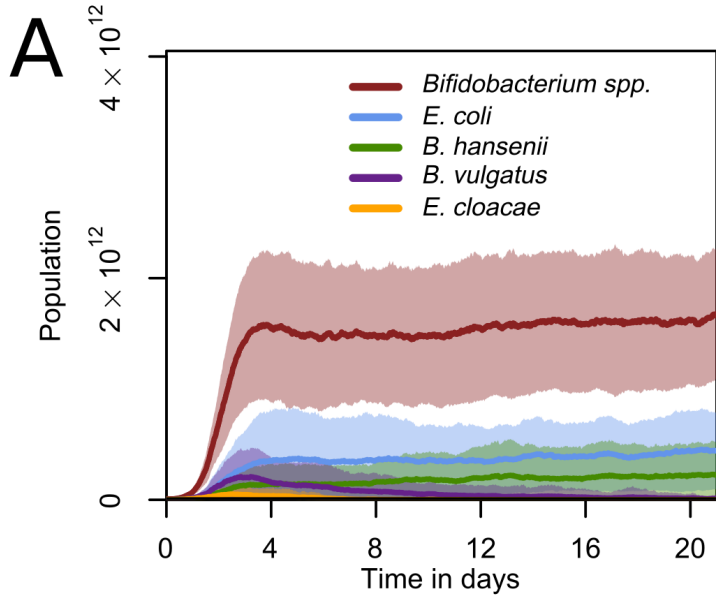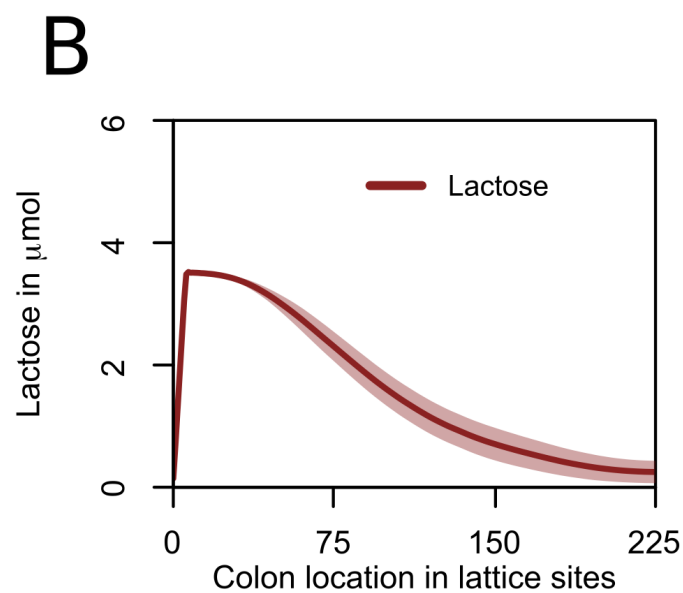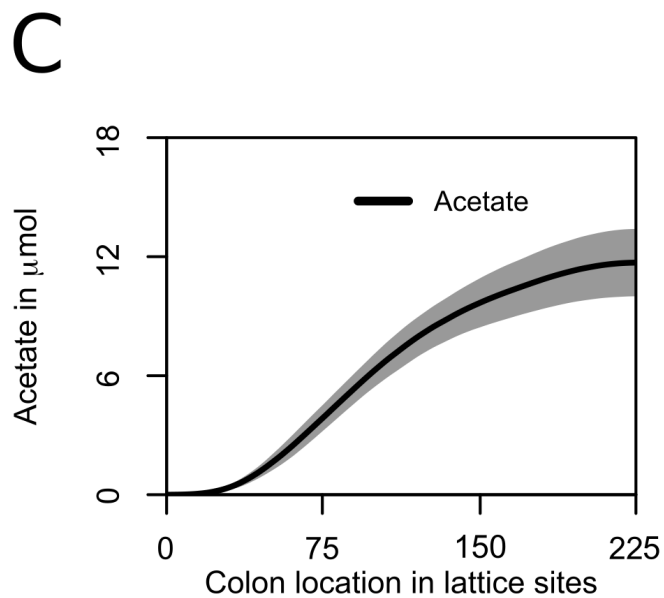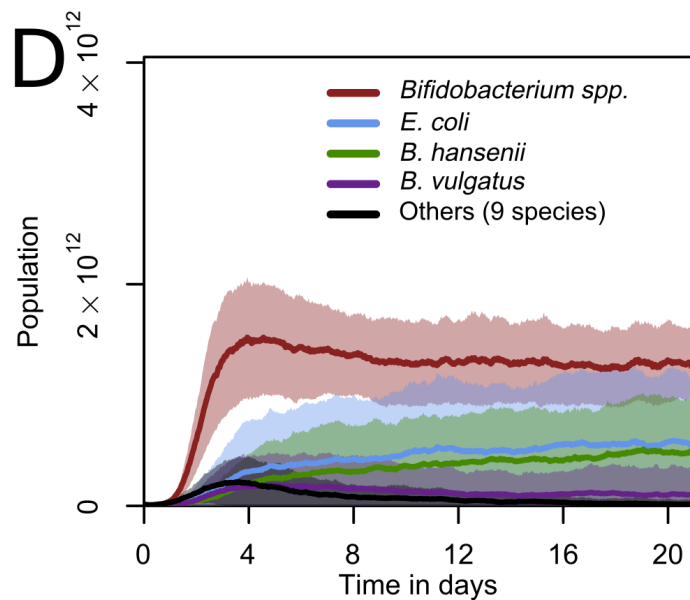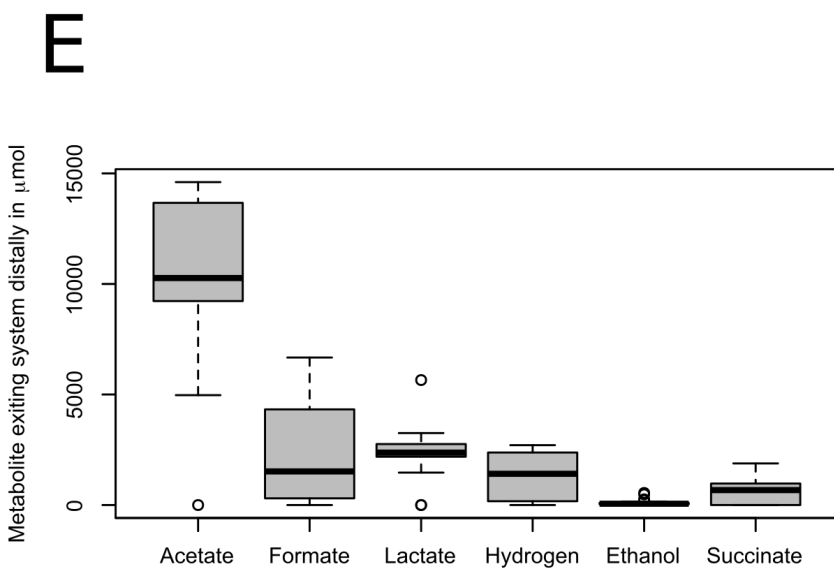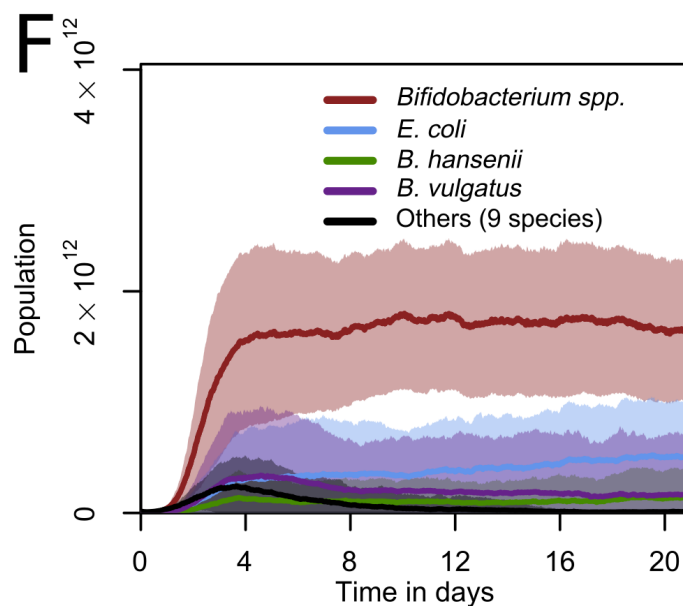

Supplement: FIG S3 [file msystems.00446-22-s0007.pdf]

**A**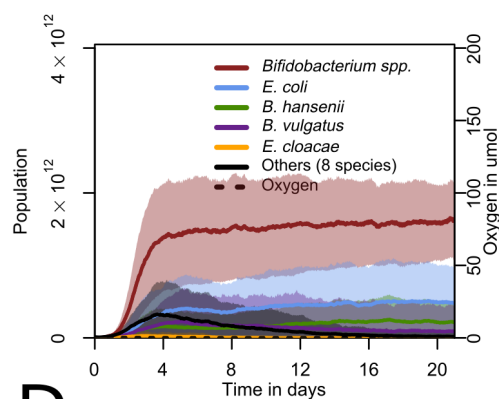**B**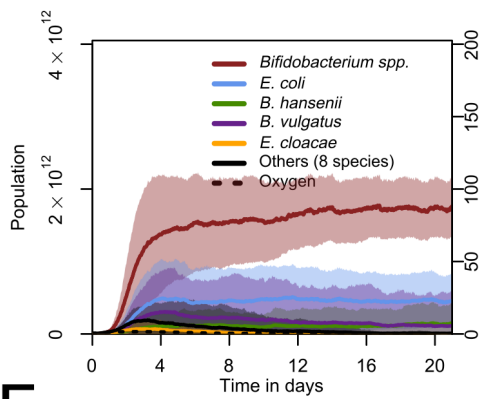**C**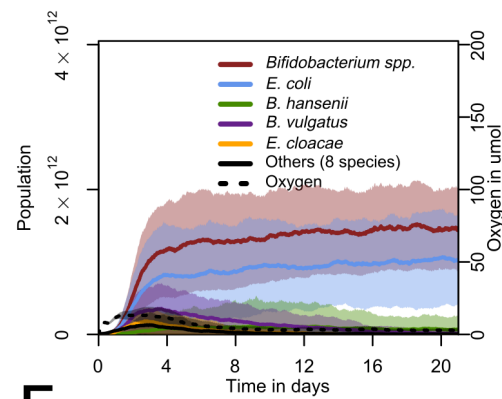**D**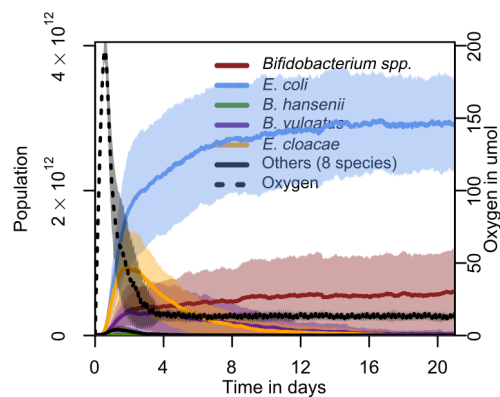**E**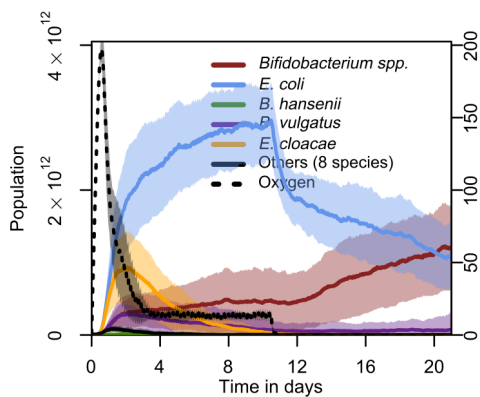**F**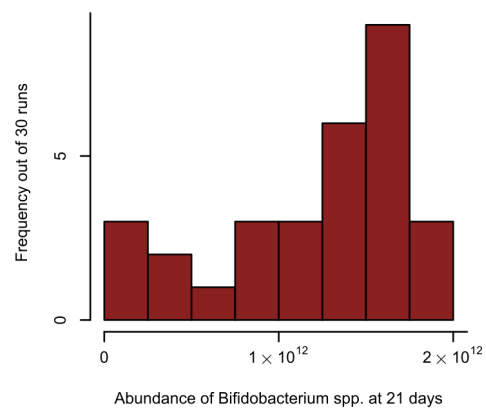**G**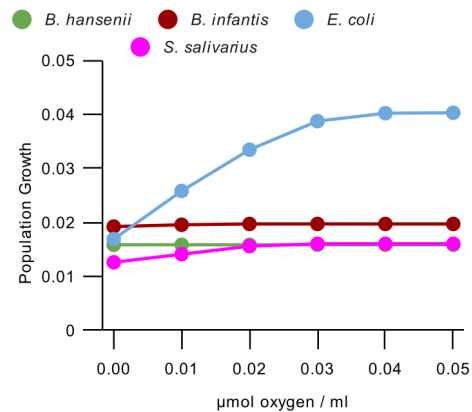**H**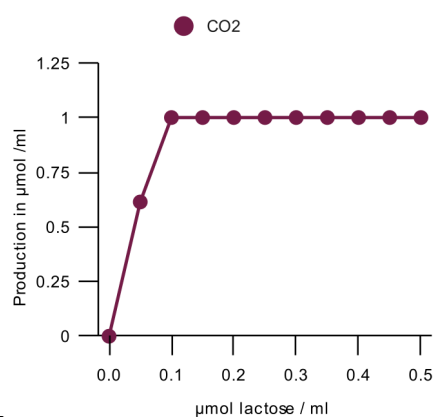**I**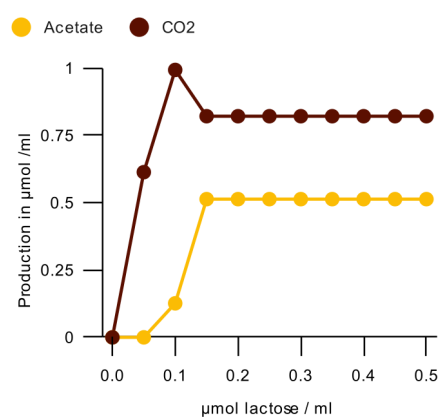**J**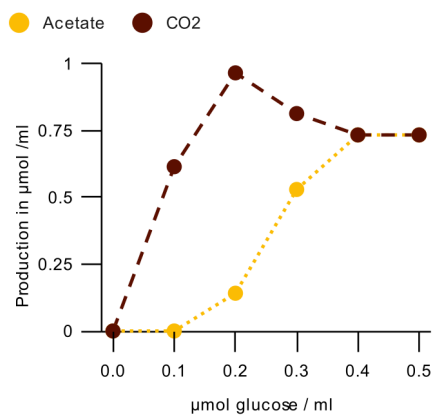**K**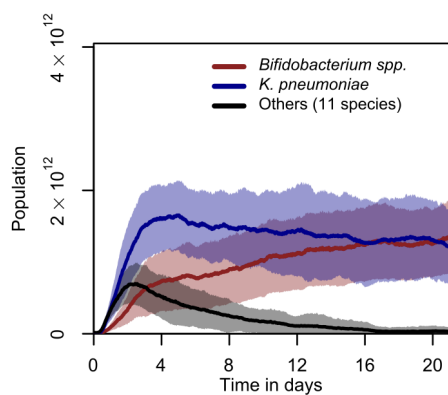**L**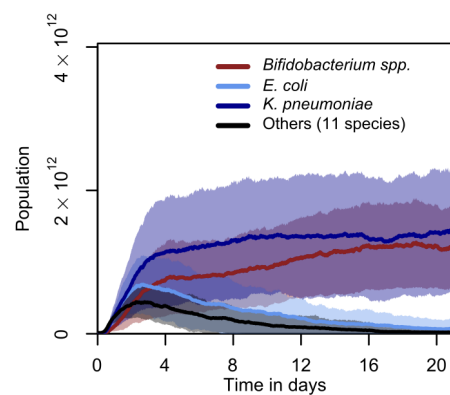

Supplement: FIG S4 [file msystems.00446-22-s0008.pdf]

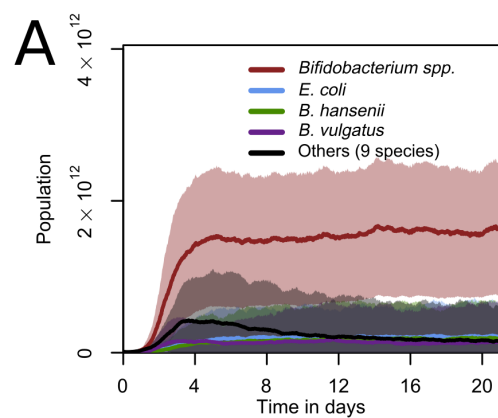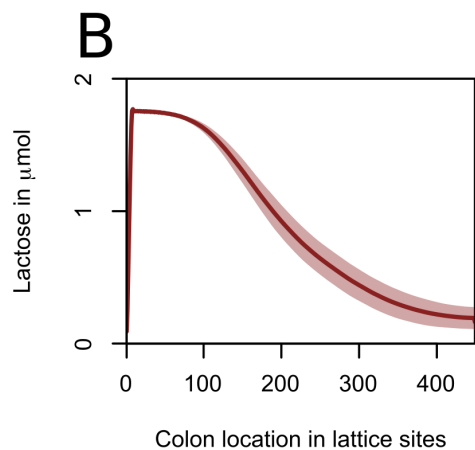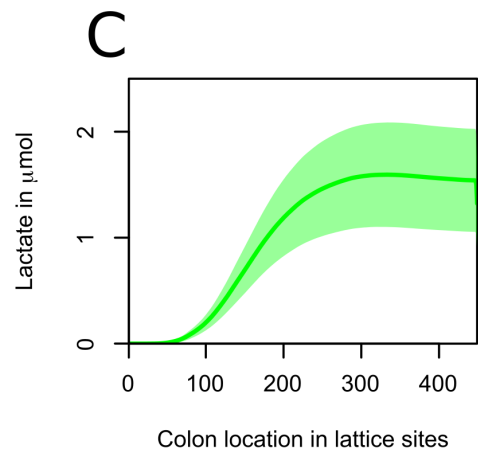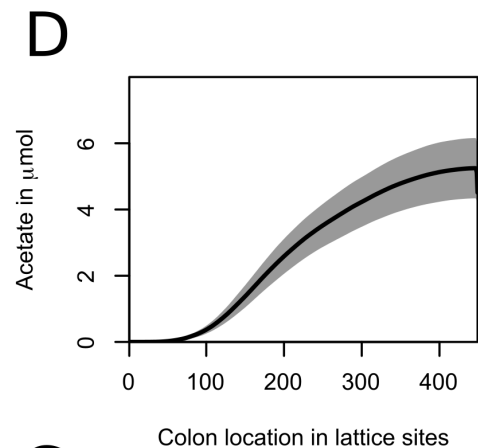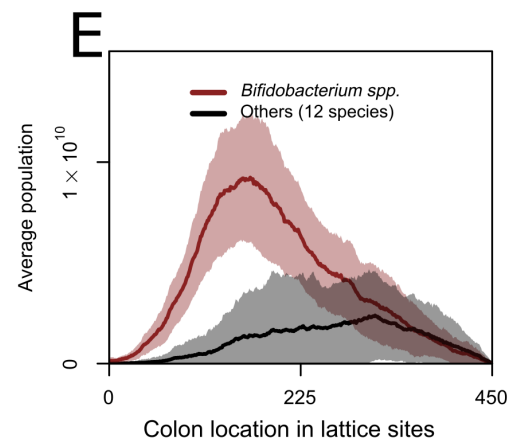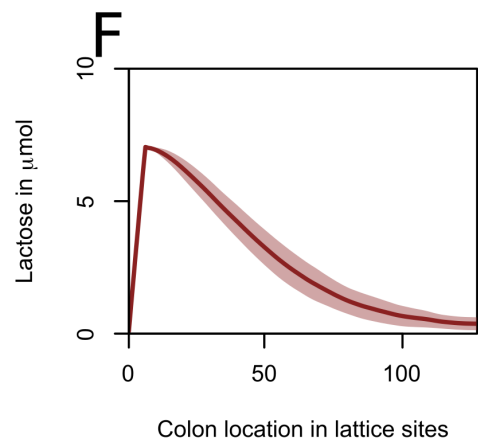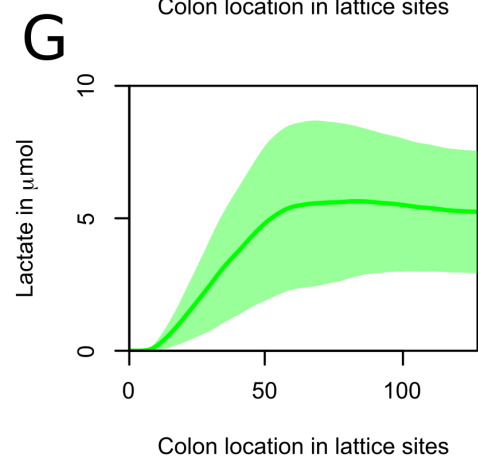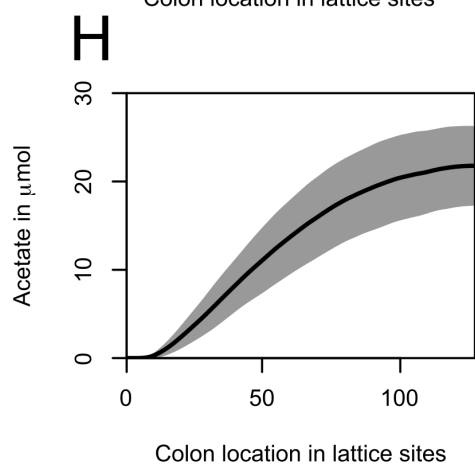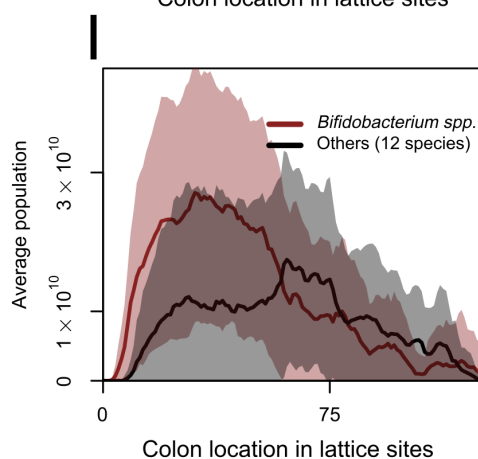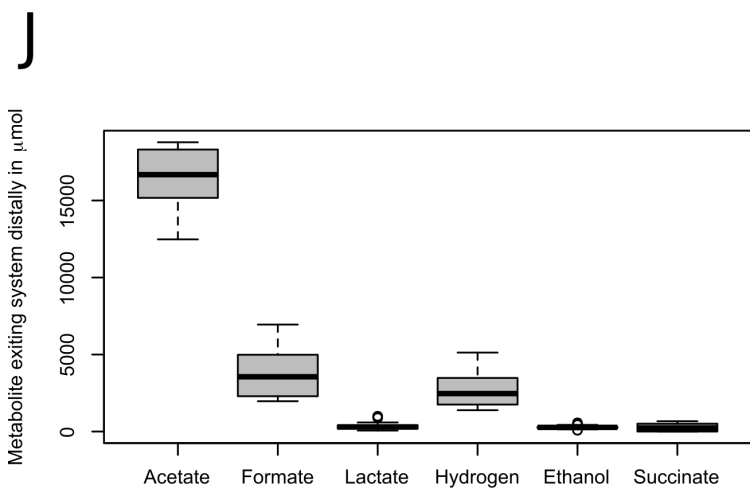

Supplement: FIG S5 [file msystems.00446-22-s0009.pdf]
